# Bilateral upper extremity myositis after COVID-19 vaccination

**DOI:** 10.1259/bjrcr.20220002

**Published:** 2022-09-12

**Authors:** Ivan Rodrigues Barros Godoy, Tatiane Cantarelli Rodrigues, Abdalla Youssef Skaf

**Affiliations:** 1 Department of Radiology, Hospital do Coração (HCor) and Teleimagem, São Paulo, Brazil; 2 Department of Diagnostic Imaging, Universidade Federal de São Paulo - UNIFESP, São Paulo, Brazil

## Abstract

Vaccination adverse reactions are common and usually are represented by transitory pain and edema. We present a case of bilateral muscle edema involving shoulders and arms due to myositis following COVID-19 vaccination, and focus on the imaging findings to differentiate with other diagnosis such as infection and tumors.

## Case presentation

A 64-year-old healthy female with no prior SARS-CoV-2 infection presented with bilateral shoulder and arm pain and weakness. The patient reported no unusual movement or trauma previous to the onset of symptoms. Pain and weakness started 15 days after the first dose of COVID-19 vaccine (adenovirus-based Covishield – Oxford/Astrazeneca). The healthcare worker responsible for the application did not report any complication during the injection. During physical examination, there were no signs of skin erythema, swelling, or fever.

## Investigation

**Figure 1.**
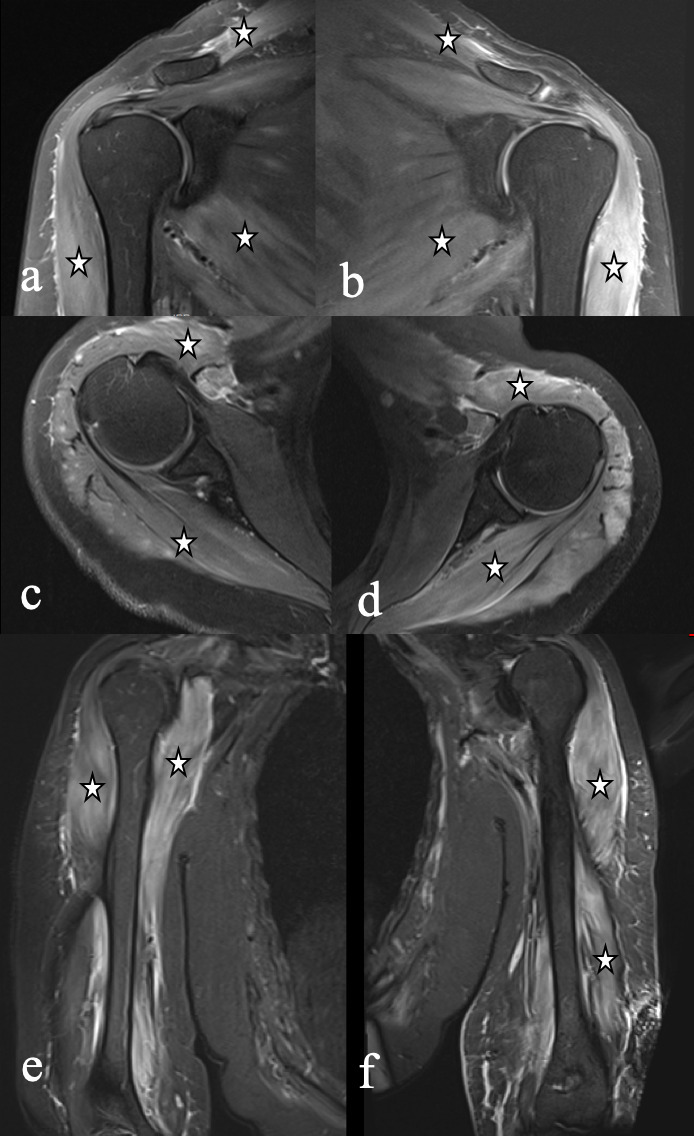
Right (**a**) and left (**b**) coronal T2 fat-saturated shoulder MR images demonstrating trapezius, deltoid, and rotator cuff muscle edema (white stars). Similar findings were observed in axial (**c, d**) coronal T2 fat-saturated shoulder MR images and coronal (**e, f**) arm MR imaging involving also biceps and triceps muscles.

Magnetic resonance (MR) of shoulders and arms was performed to evaluate arm pain and weakness demonstrating diffuse and bilateral edema involving the trapezius, deltoid, rotator cuff, biceps and triceps muscles, with no muscle tear of abscess (Figure 1).

## Differential diagnosis

Inflammatory myositis was the leading diagnosis for this case. Other potential causes of upper limb weakness such as cervical spine disc herniation, myelopathy, infection, tumors, and shoulder/arm tendinopathy were less likely given the interval appearance and imaging characteristics.

## Treatment

Patient had progressive upper extremity weakness and dysphagia and was hospitalized. Myositis was managed with rest, corticoid, and immunosuppressant therapy associated with physiotherapy.

## Outcome and follow-up

**Figure 2.**
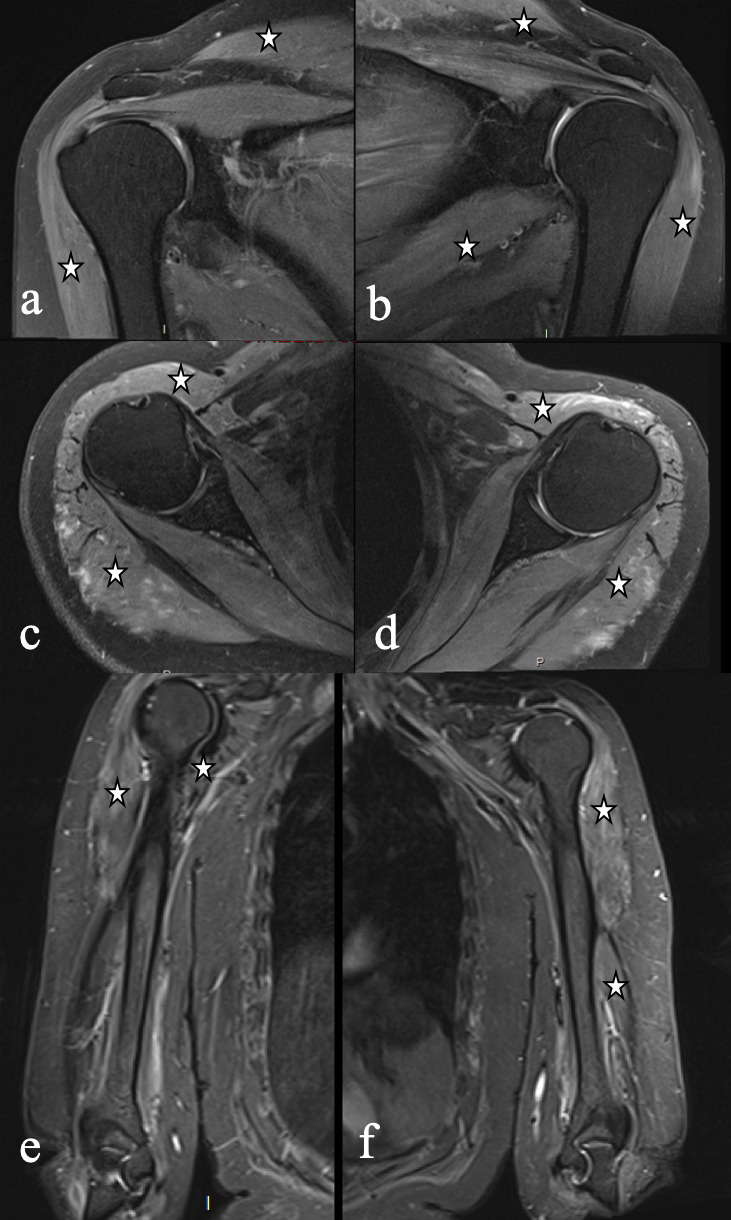
Follow-up MR imaging demonstrating reduction of muscle edema (white stars) after 4 months.

After 3 months of hospitalization, the patient regained muscle strength and was discharged. Follow-up imaging was performed 4 months after the onset of the symptoms and demonstrated bilateral muscle edema reduction without other complications (Figure 2).

Adverse reactions following vaccination are frequent and usually are characterized by transient pain, edema, and tenderness at the site of the injection.^1^ Other systemic events were described after Covishield vaccination such as fatigue, headache, body pain, fever, gastrointestinal side-effects, and even vaccine-induced immune thrombotic thrombocytopenia, raising concern for clinicians to be aware of the evaluation of those conditions. Reports identified myositis ossificans,^1^ deltoid myositis,^2^ and subacromial-subdeltoid bursitis after COVID-19 vaccination,^3^ demonstrating immune response drawn by the intramuscular injected antigen. The muscular inflammatory response may be related to the patient’s immune response to the vaccine components.^4,5^ In this report, we did not perform a muscle biopsy and the temporal link between symptoms and vaccination procedure suggests diagnosis of COVID-19 vaccine-related myositis as the cause of symptoms. Authors considered that toxic myopathy may also be a cause for reported pain, edema, and weakness. Although neuromuscular presentations of COVID-19 infection have been reported,^6^^7^bilateral upper extremity diffuse muscle edema after COVID-19 vaccination was not described before.

## Learning points

The case presented illustrates a bilateral upper extremity myositis involving shoulders and arms following COVID-19 vaccination.

Vaccination adverse reactions are common and usually are represented by transitory pain and edema.

Imaging findings are paramount to differentiate myositis with other diagnosis and key to follow-up after treatment.
